# Atrial fibrosis in a chronic murine model of obstructive sleep apnea: mechanisms and prevention by mesenchymal stem cells

**DOI:** 10.1186/1465-9921-15-54

**Published:** 2014-04-28

**Authors:** Pablo Ramos, Cira Rubies, Marta Torres, Montserrat Batlle, Ramon Farre, Josep Brugada, Josep M Montserrat, Isaac Almendros, Lluís Mont

**Affiliations:** 1 Thorax Institute, Unitat de Fibril · lació Auricular, Hospital Clínic. Universitat de Barcelona and Institut d’Investigacions Biomèdiques August Pi i Sunyer (IDIBAPS), Barcelona, Catalonia, Spain; 2 Unitat del Son. Servei Pneumologia, Hospital Clínic. Universitat de Barcelona IDIBAPS-CIBERES Barcelona, Barcelona, Catalonia, Spain; 3 Unitat de Biofísica i Bioenginyeria, Facultat de Medicina, Universitat de Barcelona-IDIBAPS-CIBERES, Barcelona, Catalonia, Spain

**Keywords:** Obstructive sleep apnea, Atrial fibrillation, Cardiac fibrosis, Mesenchymal stem cells, Animal model

## Abstract

**Background:**

OSA increases atrial fibrillation (AF) risk and is associated with poor AF treatment outcomes. However, a causal association is not firmly established and the mechanisms involved are poorly understood. The aims of this work were to determine whether chronic obstructive sleep apnea (OSA) induces an atrial pro-arrhythmogenic substrate and to explore whether mesenchymal stem cells (MSC) are able to prevent it in a rat model of OSA.

**Methods:**

A custom-made setup was used to mimic recurrent OSA-like airway obstructions in rats. OSA-rats (n = 16) were subjected to 15-second obstructions, 60 apneas/hour, 6 hours/day during 21 consecutive days. Sham rats (n = 14) were placed in the setup but no obstructions were applied. In a second series of rats, MSC were administered to OSA-rats and saline to Sham-rats. Myocardial collagen deposit was evaluated in Picrosirius-red stained samples. mRNA expression of genes involved in collagen turnover, inflammation and oxidative stress were quantified by real time PCR. MMP-2 protein levels were quantified by Western Blot.

**Results:**

A 43% greater interstitial collagen fraction was observed in the atria, but not in the ventricles, of OSA-rats compared to Sham-rats (Sham 8.32 ± 0.46% vs OSA 11.90 ± 0.59%, P < 0.01). Angiotensin-I Converting Enzyme (ACE) and Interleukin 6 (IL-6) expression were significantly increased in both atria, while Matrix Metalloproteinase-2 (MMP-2) expression was decreased. MSC administration blunted OSA-induced atrial fibrosis (Sham + Saline 8.39 ± 0.56% vs OSA + MSC 9.57 ± 0.31%, P = 0.11), as well as changes in MMP-2 and IL-6 expression. Interleukin 1-β (IL-1β) plasma concentration correlated to atrial but not ventricular fibrosis. Notably, a 2.5-fold increase in IL-1β plasma levels was observed in the OSA group, which was prevented in rats receiving MSC.

**Conclusions:**

OSA induces selective atrial fibrosis in a chronic murine model, which can be mediated in part by the systemic and local inflammation and by decreased collagen-degradation. MSCs transplantation prevents atrial fibrosis, suggesting that these stem cells could counterbalance inflammation in OSA.

## Background

Patients with obstructive sleep apnea (OSA) show both a high prevalence [1] and incidence [2] of atrial fibrillation (AF). In addition, OSA has been associated with a greater risk of AF recurrence after cardioversion [3] and catheter ablation [4,5] and a worse response to antiarrhythmic drugs [6]. Despite the clear association between OSA and AF, it is not firmly established whether this association is causal or mediated by other comorbidities often present in OSA-patients, such as obesity or hypertension [7].

Atrial structural remodeling, particularly fibrosis, is a main component in the substrate predisposing to AF [8]. Atrial fibrosis predicts disease progression and treatment outcomes [9]. It is known from murine models that exposure to recurrent airway obstructions promotes early myocardial inflammation leading to myocardial apoptosis at mid-term [10]. However, it remains unknown whether chronic exposure to recurrent apneas can reach to develop atrial fibrosis, thus explaining the higher prevalence and incidence of AF observed in OSA patients. In addition, cell-based therapies emerge as an attractive alternative to classic pharmacological treatments for the prevention of such remodeling, thereby reducing AF occurrence and progression. Among the options available for cell therapy, bone marrow mesenchymal stem cells (MSC) appear as a promising source of stem cells because of their multi-lineage potential, anti-inflammatory effects [11,12], ability to escape detection by the host immune system, and a relative ease of expansion in culture [13,14]. Recent studies have shown that MSCs attenuate cardiac fibrosis in a variety of experimental settings [15-17]. Although the knowledge on the therapeutic role of MSC in OSA models is very limited [18], there is evidence that stem cells possess anti-inflammatory properties that mitigate the early inflammatory response [11].

The aim of our study was 1) to describe OSA-induced atrial remodeling in a chronic murine model, 2) to analyze the putative mechanisms involved and 3) to investigate whether MSC have the potential to prevent such remodeling in the same OSA model.

## Methods

### Experimental sleep apnea model

This study conformed to European Community (Directive 86/609/EEC) and Spanish guidelines for the use of experimental animals and was approved by the Animal Research Ethics Committee of the University of Barcelona.

**Figure 1:**
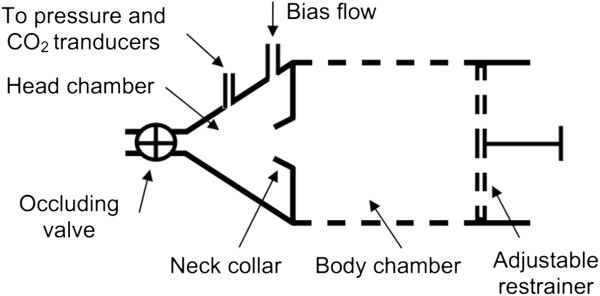
**Experimental obstructive sleep apnea (OSA) setup.** Diagram of the experimental setup to noninvasively apply recurrent airway obstructions in the rat. See text for explanation.

A chronic model of OSA previously validated by our group was used [19]. The model was designed to apply recurrent airway obstructions with an OSA pattern. Briefly, it was based on a custom-made setup consisting of 2 chambers (to fit the body and head) separated by a latex neck collar (Figure 1). The head chamber had a conical shape and was built small enough to contain the minimum possible air volume when the rat was in place. The rat breathed room air through an orifice at the vertex of the conical head chamber. A valve was placed at the entrance of the head chamber, allowing for the closure of the orifice. The valve was electronically controlled to produce intermittent obstructions mimicking those that characterize OSA, resulting in increased breathing efforts, oxygen desaturations and intermittent hypercapnia. To ensure the development of obstructive apneas, the head chamber was connected to air flow, pressure and CO_2_ transducers; pulse oximetry was measured at the rat tail. These parameters were continuously monitored by an experimented researcher who certified effective apneas (i.e., no air flow, pressure swings, increased CO_2_ and decreased pulse oximetry).

The first part of the study was carried out in 30 Sprague–Dawley male rats (250–300 g and 8 weeks old at the beginning of the experiment) randomized into 2 groups: OSA and Sham. OSA-rats (n = 16) were subjected to 15-second obstructions at a rate of 60 per hour, 6 hours per day during 21 consecutive days. Sham-rats (n = 14) were placed in the setup during the same period of time (21 days, 6 hours per day), but no obstructions were applied. Rats in both groups were progressively adapted to the experimental setting by increasing the time within the OSA/Sham device at a rate of 1 hour each day, up to 6 h at the end of the first week. Rats were housed in a controlled environment (12/12-hour light/dark cycle) and fed rodent chow and tap water *ad libitum*.

### Mesenchymal stem cells infusion

To evaluate the effect of MSC in OSA-induced structural remodeling, 8 rats undergoing the previously described OSA protocol were infused with MSC (OSA + MSC group). Seven Sham rats receiving saline vehicle (Sham + S group) were used as controls in this experiment.

MSC were obtained by culturing well-characterized Lewis rat marrow stromal cells kindly provided by the Tulane Center for Gene Therapy (New Orleans, LA, USA). The cells were cultured in MEM-alpha medium with glutamine and without ribonucleosides or deoxyribonucleosides (GIBCO, Gaithersburg, MD, USA), supplemented with 20% fetal bovine serum (HyClone Cell Culture, Cultek, Madrid, Spain), 1% antibiotic–antimycotic (containing 10,000 U/mL Penicillin G sodium; 10,000 μg/mL Streptomycin sulphate; 25 μg/mL Amphotericin B as Fungizone [GIBCO, Gaithersburg, MD, USA]) and 2% l-glutamine (GIBCO, Gaithersburg, MD, USA). The cells were grown at 37°C, 5% CO2, 100% humidity. Subconfluent cells were dissociated with 0.25% trypsin and 1 mM Ethylene Diamine Tetraacetic Acid (EDTA) in Hanks’ Balanced Salt Solution (GIBCO, Gaithersburg, MD, USA) and subcultured at low density in new culture flasks. To prepare the injection, MSC were trypsinized and 5 × 10^6^ cells were suspended in 500 μL of Phosphate Buffered Saline (GIBCO, Gaithersburg, MD, USA). This cell preparation was slowly delivered (30 seconds) to the rat through the penile vein the first day of apneas application and every 4 days thereafter. Rats were anaesthetized with short-acting inhaled isoflourane (2%) before and during every injection.

All rats in the four experimental groups (Sham, Sham + S, OSA, OSA + MSC) were carefully inspected daily to monitor animal well-being. No rats showed any signs of stress or MSC-related side effects and thus, no rats needed to be excluded from the analysis.

### Sample collection and RNA isolation

Once anesthetized with intraperitoneal urethane 10% (1 g/kg), animals were sacrificed by exsanguination through carotid artery cannulation. Blood samples were collected in EDTA tubes and centrifuged for 15 minutes at 3000 rpm. Plasma was collected and frozen at -80°C.

Hearts were quickly removed and weighted, and tissue samples were obtained from the left ventricle (LV) free wall, right ventricle (RV) free wall, and both atrial appendages: right atrium (RA) and left atrium (LA). Samples were snap-frozen in liquid nitrogen for posterior molecular biology analysis. For histological studies, transversal sections from the midventricular and basal (including both atria) regions of the heart were obtained, fixed in formol 3% for 24 hours and embedded in paraffin.

Total RNA was isolated from the 4 cardiac chambers. Myocardial tissue was first homogenized with Trizol® reagent (Life Technologies) and further purified with chloroform (C-2432, Sigma-Aldrich). Final RNA was obtained with silica columns (RNA Aqueous kit, Ambion) according to the manufacturer's protocol. The integrity of the resulting RNA was assessed in formaldehyde-denaturing agarose gels. A reverse transcription protocol with random primers was applied to 1 μg of total RNA for cDNA synthesis with the addition of RNAse inhibitors (High capacity cDNA RT kit, Life Technologies, CA, USA) with a MJ Research PTC 200 thermal cycler (MJ Research, MA, USA).

### Real time polymerase chain reaction (real time PCR)

Messenger RNA (mRNA) expression of selected key-player genes in the collagen synthesis and degradation balance was measured in the four cardiac chambers. Genes involved in collagen-synthesis promotion (ACE, TGF-β1), collagen maturation and cross-linking (LOX), and degradation (MMP-2, MMP-3, MMP-9, MMP-10, TIMP-1, TIMP-2), as well as oxidative stress (eNOS) and inflammation (IL-6) were quantified. mRNA expression was assayed with a real-time PCR 7900 thermal cycler (AB, Applied Biosystems, CA, USA) using TaqMan Universal PCR Master Mix with AmpErase UNG. Specific TaqMan single-gene expression assays are shown in Table 1. All mRNA quantification results are shown relative to a cDNA pool with sequential dilutions and units are given as ng-equivalents of cDNA (ng-equ).

**Table 1:** TaqMan one-gene expression assays

**Gene**	**Abbreviation**	**TaqMan reference**
Angiotensin-I Converting Enzyme	ACE1	Rn00561094_m1
Transforming Growth Factor-β1	TGF-β1	Rn00572010_m1
Lysyl Oxidase	LOX	Rn01491829_m1
Matrix Metallopeptidase-2	MMP-2	Rn01538170_m1
Matrix Metallopeptidase-3	MMP-3	Rn00591740_m1
Matrix Metallopeptidase-9	MMP-9	Rn00579162_m1
Matrix Metallopeptidase-10	MMP-10	Rn00591678_m1
Tissue Inhibitor of Metallopeptidase-1	TIMP1	Rn00587558-m1
Tissue Inhibitor of Metallopeptidase-2	TIMP2	Rn00573232_m1
Nitric Oxide Synthase III	NOS3	Rn02132634_s1
Interleukin-6	IL-6	Rn01410330_m1

### Protein isolation and analysis by western blot

Myocardial proteins were extracted from each heart cavity in all experimental groups. Samples were submerged in 0.5 mL of ice-cold protein lysis buffer containing: RIPA buffer (R0278, Sigma), 1 mM phenylmethanesulfonyl fluoride (P7626, Sigma), 1 mM sodium orthovanadate (S6508, Sigma), 1 mM Pefabloc (11429868001, Roche) and complete Mini Protease Inhibitor Cocktail (11836153001, Roche). Samples were homogenized with an Omni TH homogenizer (Omni International Inc.). After 1 hour of rotation at 4°C, samples were centrifuged at 10.000 g at 4°C for 30 minutes. The upper phase was collected and the total protein concentration was quantified with the Pierce BCA protein Assay method (23227, Thermo Scientific, Pierce) relative to a BSA standard curve. Forty μg of total protein extract were loaded with a reducing buffer (2.5% of β-mercaptoethanol) to NuPage® 4–12% Bis-Tris Gel (NP0322). A western blot was performed with the Novex® gels methodology (Invitrogen). Proteins were transferred to a nitrocellulose membrane using a blot gel transfer (IB3010-01) and the iBlot® Dry Blotting System. Proper transfer was checked by Ponceau staining. After 1 hour blockade of the membrane with phosphate buffered saline solution (PBS, Fisher Scientific), 0.1% Tween 20 (P1379, Sigma-Aldrich) and 5% of skimmed milk, it was incubated overnight at 4°C with the MMP-2 primary antibody (ab37150, Abcam) diluted 1/500. Afterwards, the membrane was incubated during 1 hour with an HRP-Goat anti rabbit secondary antibody diluted 1/1000 (31460 Thermo Scientific). Final detection of the MMP-2 protein bands was accomplished with the ECL kit Supersignal West Pico Chemioluminescent Substrate (34080, Thermo Scientific).

Bands around 60 KDa for MMP-2 (in antibody and ECL incubated membranes) and around 40 KDa (in Ponceau-stained membranes) were quantified (ImageJ, NIH, Maryland, USA). Loading of each sample was normalized with the Ponceau band around 40 KDa. Results are given in arbitrary units (A.U.) as the ratio between the normalized densities of each sample divided by the normalized density of a standard loaded in each gel.

### Plasma cytokines

Systemic inflammation-related cytokines -proinflammatory and profibrotic Interleukin-1 beta (IL-1β), and anti-inflammatory Interleukin-10 (IL-10)- were measured in plasma obtained at sacrifice with Quantikine ELISA assays RLB00 and R1000, respectively (R&D Systems, MN, USA).

### CD90 immunofluorescence

A CD90 immunofluorescence assay was performed in myocardial parafine-embedded sections from rats injected with MSC, and in MSC cultured in 30-40% confluent chamber slides (positive control). Cultured MSC were fixed with 4% paraformaldehyde. Antigenic retrieval was achieved with a sodium citrate 10 mM bath at 80°C during 40 min. After blockade of unspecific unions, samples were incubated overnight with Anti-Rat CD90, FITC conjugated antibody (MR5001, Caltag, Invitrogen) diluted 1/200. Sudan Black was used to mask auto-fluorescence. Both myocardial sections and chambers were added a DAPI-containing mounting media and covered. The same staining protocol excluding Anti-CD90 antibody addition was used as a negative control for both groups.

### Myocardial fibrosis quantification

Mid-ventricular and atrial sections of paraffin-embedded tissue, 4 microns thick, were stained with Picrosirius-red as previously described [20]. Four random pictures (40×) from RV and LV free wall, and 6 random pictures (100×) from the atria were taken from each sample with an Olympus BX51 microscope and an Olympus DP50 camera (Olympus Corporation, Japan).

To estimate hypertrophy of the cardiac chambers, RV free wall, interventricular septum (IVS) and LV free wall thickness were measured in transversal sections at mid-ventricular level. The interstitial collagen fraction was assessed with a semiautomatic color-threshold detection software (AnalySIS®, Soft Imaging System, Germany). Right and left atrial collagen fraction were quantified together. Epicardial, endocardial, and perivascular fibrosis were excluded from the analysis. All measures were carried out blind by a single investigator.

### Statistical analysis

All variables followed a normal distribution (Shapiro-Wilks test) and are expressed as mean ± standard error of the mean (SEM). Grubbs test was used to exclude extreme outliers (maximum one per group). Comparisons between 2 groups were carried out with a non-paired Student’s t-test. A Pearson product–moment correlation coefficient was computed to assess the relationship between the plasmatic IL-1β levels and the myocardial collagen fraction. Statistical calculations were performed with SPSS v16.0 (SPSS Institute Inc, Cary, NC, USA). A p-value <0.05 was considered for significance.

## Results

### Obstructive sleep apnea induces atrial fibrosis

#### Histological analysis

The 3-week OSA protocol did not induce global or specific-chamber hypertrophy in rats. No significant differences between both groups were found in heart weight (Sham 1.21 ± 0.02 g. vs OSA 1.14 ± 0.03 g, P = 0.08), RV free wall thickness (Sham 938 ± 55 μm vs OSA 876 ± 65 μm, P = 0.49), IVS thickness (Sham 2234 ± 162 μm vs OSA 2069 ± 190 μm, P = 0.54) or LV free wall thickness (Sham 2427 ± 178 μm vs OSA 2300 ± 190 μm, P = 0.64).

**Figure 2:**
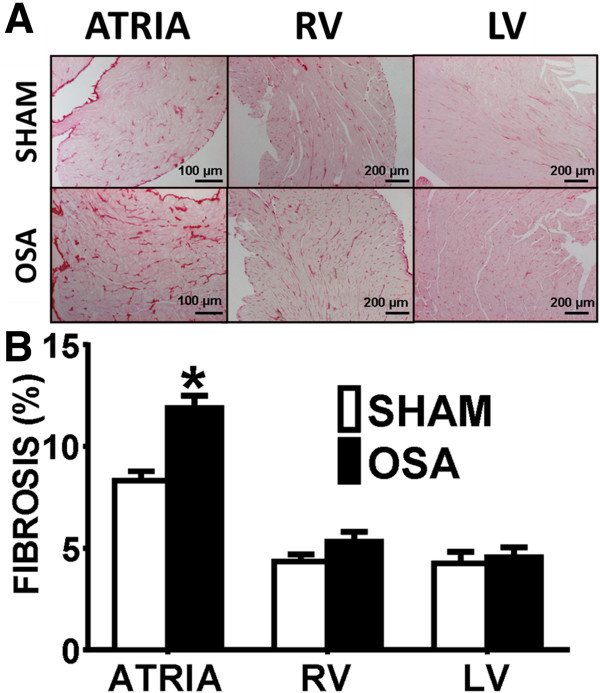
**Fibrosis assessment in OSA and Sham rats. A**. Representative Picrosirius-stained photomicrographs of atrial, right ventricle (RV) and left ventricle (LV) sections from Sham (n = 14) and OSA (n = 16) rats. Atria and ventricular samples were stained in different days, and pictures obtained at different magnifications, so results from atria and ventricles should not be compared. **B**. Quantification of collagen fraction in the atria, RV and LV. (Mean ± SEM, *P < 0.05).

We evaluated myocardial fibrosis in histological preparations. Figure 2A shows representative photomicrographs of Picrosirius-red stained LV, RV and atrial sections from Sham and OSA rats. OSA induced a significant 43% higher atrial interstitial collagen deposition as compared to the sham group (Figure 2B). Increased fibrosis deposition was diffuse and homogenous, with no patchy fibrotic infiltrates in any of the analyzed samples. No differences in collagen deposit density were observed between OSA and Sham groups in RV and LV free wall.

#### mRNA expression and protein synthesis

**Figure 3:**
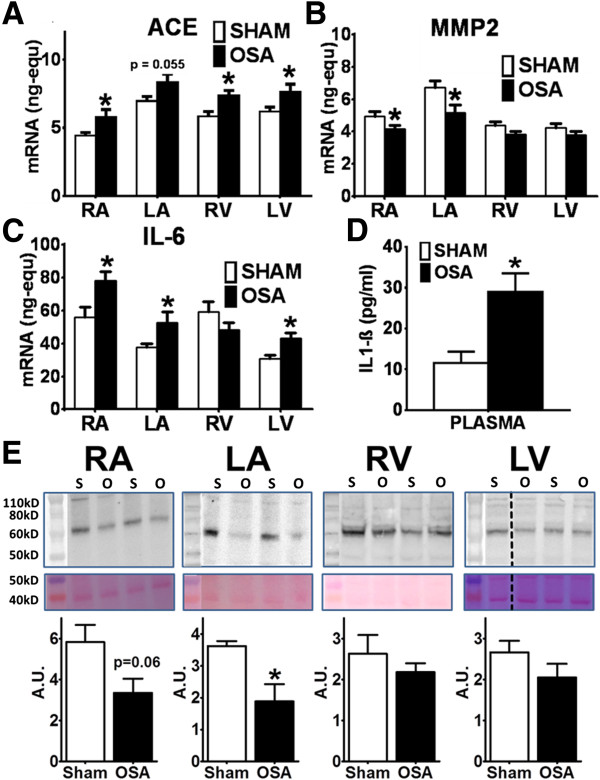
**Fibrosis mechanisms assessment in OSA and Sham rats. (A)** ACE, **(B)** MMP-2 and **(C)** IL-6 mRNA expression (ng-equ) in the four cardiac chambers of Sham (n = 14) and OSA (n = 16) rats (Mean ± SEM, *P < 0.05). **D**. Plasmatic concentration (pg/mL) of IL-1β in Sham and OSA (Mean ± SEM, *P < 0.05). **E**. MMP-2 protein levels quantification. Representative MMP-2 blots (upper panel), Ponceau-stained membranes (middle panel) and normalized quantification (lower panel) (Mean ± SEM, *P < 0.05). n = 4-6/group. Left lane is a molecular-weight marker lane; the picture was obtained with visible light. Dashed line means non-contiguous lane. A.U.: Arbitrary Units.

**Figure 4:**
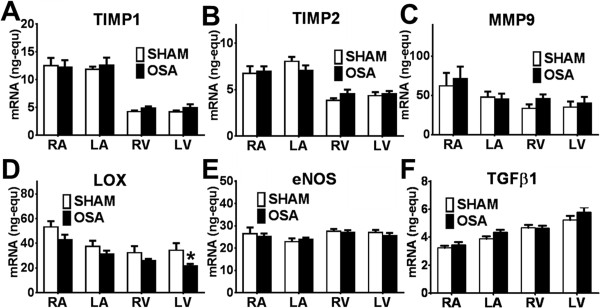
**mRNA expression in the OSA and Sham rats. (A)** TIMP-1, **(B)** TIMP-2, **(C)** MMP-9, **(D)** LOX, **(E)** eNOS and **(F)** TGF-β1 mRNA expression (ng-equ) in the four cardiac chambers of Sham (n = 14) and OSA (n = 16) rats (Mean ± SEM, *P < 0.05).

To study the mechanisms of myocardial fibrosis, we quantified mRNA expression of genes involved in myocardial collagen turnover, inflammation and oxidative stress in the 4 cardiac chambers of sham and OSA-rats. ACE expression was significantly greater in the RA, RV and LV, and was close to significance (p = 0.055) in the LA, of OSA-rats compared with Sham-rats (Figure 3A). Expression of collagen promoters TGF-β1 and LOX was unaltered (Figure 4D, F). Regarding collagen degradation, MMP-2 expression was significantly decreased in both atria, but not in the ventricles (Figure 3B). No significant changes were found in any other metalloproteinase or metalloproteinase inhibitor (Figure 4A-C). MMP-3 and MMP-10 levels were undetectable and are not shown. Finally, IL-6 expression was significantly higher in both atria and in the LV of the OSA group, but showed no differences in the RV (Figure 3C).

As a potential regulator of myocardial fibrosis in our model, protein levels of MMP-2 were analyzed in the four cardiac chambers. Representative blots and quantification are shown in Figure 3E. MMP-2 protein levels were lower in left atria from OSA-rats than in left atria from Sham-rats (p = 0.03), and showed borderline significance for the right atrium (p = 0.06). No differences were found between Sham and OSA groups in the left and right ventricles.

#### Plasma cytokines

**Figure 5:**
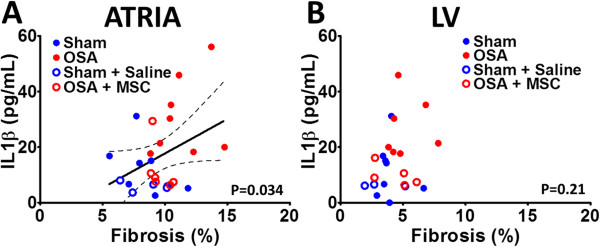
**Correlation between plasma IL-1β and myocardial fibrosis at the atrial (A) and ventricular (B) level.** Dashed line means 95% confidence interval.

OSA-induced changes in systemic inflammation were studied in plasma samples. A remarkable 2.5-fold increase in pro-inflammatory IL-1β plasma levels was observed in the OSA group compared to the sham group (Figure 3D). IL-1β plasma levels positively correlated to atrial (r = 0.404, p = 0.037, Figure 5A), but not ventricular (p = 0.21, Figure 5B) collagen fraction. Conversely, no differences were found in IL-10 plasma levels between both groups (Sham 11.25 ± 2.96 pg/mL vs OSA 7.85 ± 1.41 pg/mL, P = 0.26). Plasma IL-10 did not correlate to either atrial or ventricular collagen fraction (results not shown, p = 0.209 and p = 0.373, respectively).

### Mesenchymal stem cells prevent sleep apnea-induced atrial fibrosis

#### Histological analysis

**Figure 6:**
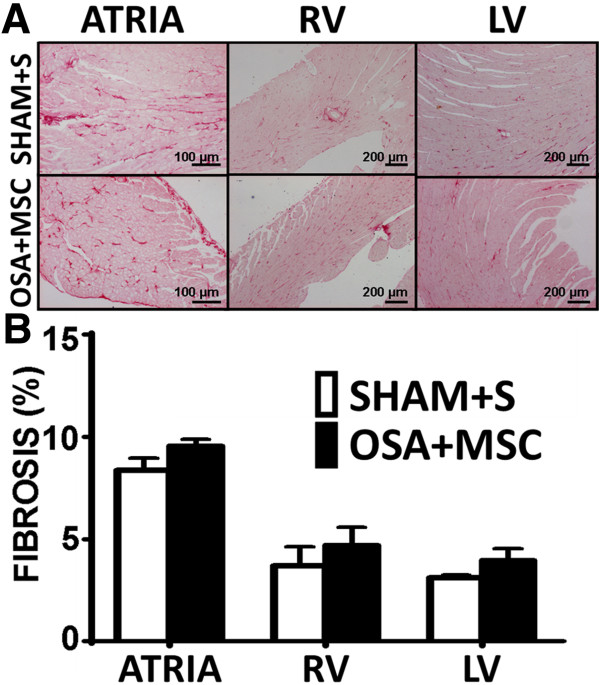
**Fibrosis assessment in OSA + MSC and Sham + S rats. A**. Picrosirius-stained photomicrographs of atrial sections, right ventricular (RV) sections and left ventricular (LV) sections from Sham + Saline (n = 7) and OSA + MSC (n = 8) rats. Atria and ventricular samples were stained in different days, and pictures obtained at different magnification, so results from atria and ventricles should not be compared. **B**. Quantification of collagen fraction in the atria, RV and LV measured in Picrosirius-red stained samples (Mean ± SEM).

As in the first set of rats, no differences between OSA + MSC and Sham + S groups were found in heart weight nor in the ventricular collagen fraction. OSA-induced atrial fibrosis was prevented by the administration of MSC; OSA + MSC and Sham + S groups showed a similar atrial collagen fraction (Figure 6).

#### CD90 immunofluorescence

**Figure 7:**
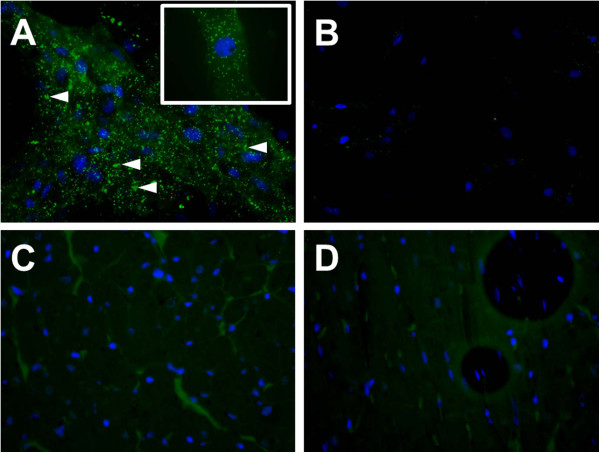
**CD90 immunofluorescence myocardial stained samples. A**. Cultured MSC, positive control for CD90 (40x). 100x augmentation in the upper box. Abundant CD90 deposits (some marked with arrowhead) are present. **B**. Cultured MSC, negative control (x40, no anti-CD90 antibody added). **C**. Sample of interest (left atrium), CD90 stained. No positive cells are marked (x40). **D**. Sample of interest, negative control (x40, no anti-CD90 antibody added). Blue: nuclei (DAPI); green: CD90.

We used the sensitive MSC-marker CD90 to study their presence in the myocardium of OSA + MSC rats. Figure 7 shows representative microphotographies of a positive control (cultured MSC in A-B) and sample of interest (LA from OSA + MSC rats in C-D). After thoroughly exploring the myocardium samples, no CD90 positive cells were found in the atria or ventricles of five OSA + MSC rats myocardial tissue-sections.

#### mRNA expression and protein synthesis

**Figure 8:**
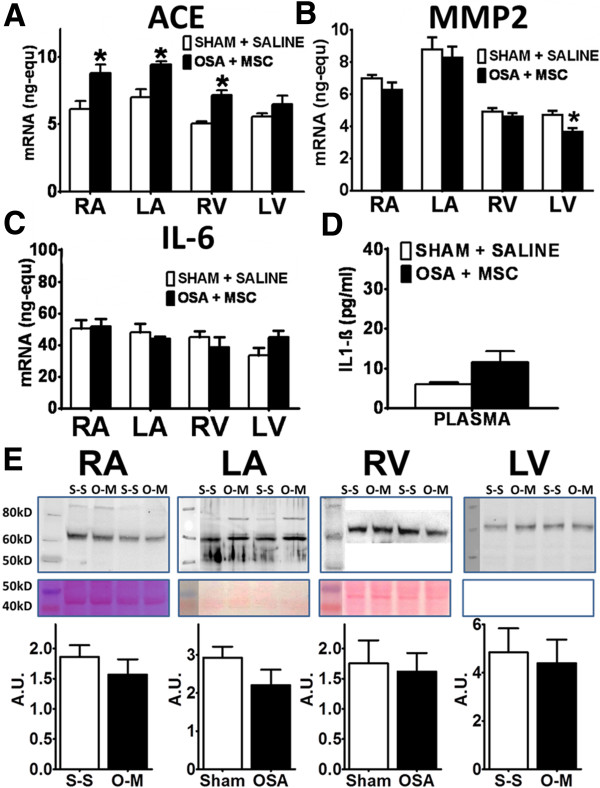
**Assessment of fibrosis mechanisms in OSA + MSC vs Sham + S rats. (A)** ACE, **(B)** MMP-2 and **(C)** IL-6 mRNA expression (ng-equ) in the four cardiac chambers of Sham + Saline (n = 7) and OSA + MSC (n = 8) rats (Mean ± SEM *P < 0.05). **D**. Plasmatic concentration (pg/mL) of IL-1β in Sham + Saline and OSA + MSC rats (Mean ± SEM *P < 0.05). **E**. MMP-2 protein levels quantification. Representative MMP-2 blots (upper panel), Ponceau-stained membranes (middle panel) and normalized quantification (lower panel) (Mean ± SEM). n = 4-6/group. Left lane is a molecular-weight marker lane; the picture was obtained with visible light. A.U.: Arbitrary Units.

**Figure 9:**
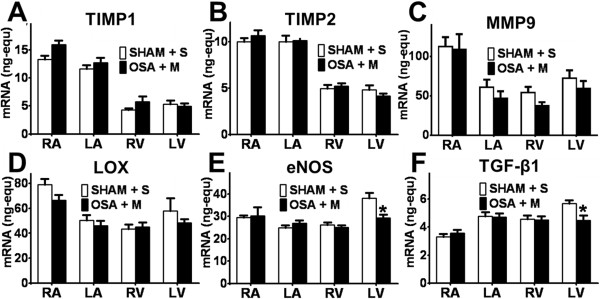
**mRNA expression in the OSA + MSC and Sham + S rats. (A)** TIMP-1, **(B)** TIMP-2, **(C)** MMP-9, **(D)** LOX, **(E)** eNOS and **(F)** TGF-β1 mRNA expression (ng-equ) in the four cardiac chambers of Sham + Saline (n = 7) and OSA + MSC (n = 8) rats (Mean ± SEM *P < 0.05).

Beneficial effects of MSC infusion in the atria of OSA rats were not accompanied by regression of changes in ACE mRNA expression. ACE mRNA expression remained higher in the RA and LA of OSA + MSC rats compared with Sham + S rats (Figure 8A). Conversely, MSC blunted OSA-induced atrial MMP-2 downregulation; MMP-2 expression was similar in OSA + MSC and Sham + S groups (Figure 8B). IL-6 OSA-induced increase was reverted after MSC-infusion (Figure 8C). No differences were observed in the expression of the other genes analyzed in the atria, though changes of uncertain significance were found for MMP-2 (Figure 8B), eNOS and TGF-β1 in the LV (Figure 9).

Results for MMP-2 mRNA expression in the atria were confirmed in protein levels quantification (Figure 8E). No differences were found in MMP-2 protein levels between Sham + S and OSA + MSC rats in the left or right atria. Contrary to mRNA expression, no differences were seen in MMP-2 protein levels in the LV (Sham + S vs OSA + MSC).

#### Plasma cytokines

Figure 8D shows mean IL-1β levels in the Sham + S and OSA + MSC groups. OSA-induced increase in IL-1β plasma levels was blunted in the OSA group receiving MSC.

## Discussion

In the present work, we have demonstrated for the first time that chronic exposure of rats to recurrent apneas can promote atrial fibrosis, an established substrate for AF. This process seems to be mainly mediated through an increased local and systemic inflammation and a reduced collagen-degradation. Also, our data revealed that the OSA-induced fibrosis can be prevented by the infusion of MSC.

The application of intermittent hypoxia by modifying oxygen concentration in breathed gas, which is the most widely used model in OSA, is able to induce systemic inflammation. However, the setting employed in our work, in addition to intermittent hypoxia, allowed the application of increased negative intrathoracic-pressure swings [19]. These increased inspiratory efforts could aggravate the early local inflammatory response triggered by intermittent hypoxia alone. In addition, both factors could participate in the development of AF by different mechanisms; i.e. intermittent hypoxia can elevate the systemic blood pressure but also, increased intrathoracic pressure swings can increase left ventricular transmural pressure [21] and may lead to repetitive atrial stretch which chronically could lead to left atrial enlargement and fibrosis.

Previous experimental studies reported enhanced AF-inducibility in acute OSA models, likely mediated by imbalances in the autonomic system [22,23]. Iwasaki et al. [24] suggested a role for transient LA distension in AF substrate in an acute OSA model in obese rats. Few experimental works have studied the effects of OSA in cardiac structure at the histological level. Simpson et al. [25] showed that intermittent respiratory occlusions acutely induce multifocal areas of necrosis in both ventricles, and Chen et al. [26] described LV myocyte hypertrophy and apoptosis in a chronic intermittent hypoxia model. Neither of these two works studied atrial remodeling. To the best of our knowledge, our work is the first to describe the development of an arrhythmogenic structural substrate in the atria in a chronic OSA model.

Chamber-specific myocardial fibrosis (selective atrial fibrosis while preserving the ventricles) has been observed in our and other experimental settings [20,27-29]. Various mechanisms explain increased atrial susceptibility to fibrosis. Differential stretch and mechanical-loading properties between the atria and ventricles [30], differences in atrial- and ventricular-fibroblast reactivity [31], and increased fibrotic response to myocardial ACE activity in the atria [32] have been reported and may contribute to atrial fibrotic susceptibility in our model.

**Figure 10:**
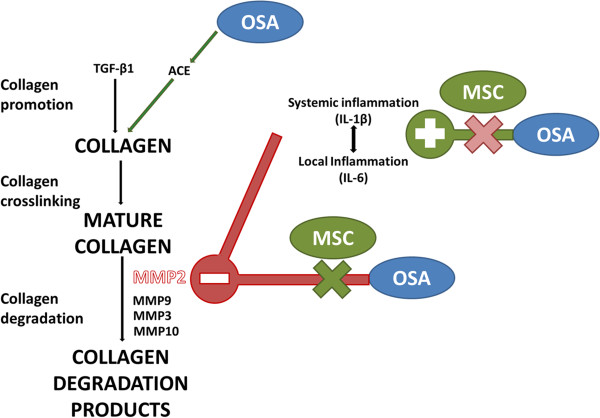
**Proposed pathophysiology of OSA-induced atrial fibrosis and MSC mechanism of action.** OSA acts as a pro-inflammatory stimulus and inhibits MMP-2 synthesis, reducing collagen degradation and thus favoring collagen accumulation. MSC administration blunts inflammation and normalizes MMP-2 synthesis, thereby increasing collagen degradation and preventing from collagen deposit. OSA also increases ACE expression, but its role in fibrosis promotion is uncertain. Red lines represent inhibition, green lines represent activation. MSC: mesenchymal stem cells. OSA: obstructive sleep apnea.

The mechanisms by which OSA induces atrial fibrosis are unknown. Our results are summarized in Figure 10 and yield mechanistic insights. Myocardial collagen content is critically dependent on the balance between synthesis and degradation. In the present OSA-model, both ACE upregulation [32] and MMP-2 downregulation [33] could promote myocardial fibrosis. Nevertheless, further data from our work suggest that MMP-2 is central in OSA-induced atrial fibrosis, while the role of ACE is negligible. First, ACE expression was increased in all cardiac chambers in the OSA-group, but increased fibrosis was only found in the atria. Remarkably, MMP-2 was selectively downregulated in the atria. Second, TGF-β1, an important fibrotic mediator and an ACE downstream mediator, was not increased in OSA-rats. Results after infusion of MSC further emphasize a critical role for MMP2. OSA-rats in the MSC-treated group had an atrial collagen fraction similar to the Sham + S group. This finding was accompanied by a normalization in MMP-2 synthesis, likely leading to a higher extracellular matrix degradation activity and hence, reduced fibrosis. Notably, although MSC infusion prevented atrial fibrosis, it was not able to prevent OSA-induced ACE-increase, thus suggesting that increased ACE expression by itself is not enough to sustain OSA-induced atrial fibrosis.

The expression of the remaining collagen degradation (MMP-3, MMP-9, MMP-10, TIMP-1, TIMP-2) and collagen cross-linking (LOX) mediators were not affected in our model.

Besides a profibrotic effect, a growing core of evidence points to OSA as a pro-inflammatory disease [34]. Previous work by our group showed that OSA induces a systemic inflammatory response in an acute animal model. This response was characterized by a significant increase in IL-1β plasmatic levels that was prevented by MSC infusion [11]. The results of the present work confirm this finding in a chronic OSA model and show an increase in the pro-inflammatory cytokine IL-6 in both atria and the LV. Inflammation and fibrosis are closely linked, with several interrelated metabolic pathways. For example, IL-6 is involved in the development of myocardial fibrosis in other experimental settings [35]. Moreover, plasma IL-1β selectively correlated to atrial fibrosis intensity, further highlighting higher fibroblast reactivity in the atria than in the LV [31]. Interestingly, both systemic (IL-1β) and local (IL-6) inflammatory responses were prevented by MSC infusion. Notably, a growing core of evidence is suggesting that an anti-inflammatory effect underlies beneficial effects of MSC [12,36].

Bone marrow MSC properties including pluripotency, avoidance of detection by the host immune system, and ease of expansion in culture make them an attractive option for cell therapy [13,14]. Several studies have shown that MSC transplantation significantly decreases fibrosis in the heart [16,17], lung [37], kidney [38], skin [39] and liver [40]. Although the mechanisms whereby MSCs reduce tissue fibrosis remain unclear, previous studies support our finding that MSC-induced MMP-upregulation might be a hallmark of their antifibrotic effect. In mice skin, Wu et al. [39] demonstrated an antifibrotic effect of bone marrow MSC that was partially mediated by an increased MMP-2 collagen-degradation. Mias et al. [15] showed that MSC injection reduced ventricular fibrosis in a rat model of myocardial infarction by stimulating MMP-2 and MMP-9 secretion in fibroblasts. Consistent with a central role of MMP-2, MSC collagen-accumulation prevention was lost in MMP-2 knock-out fibroblasts [15]. This effect is probably mediated by secreted factors in a paracrine/endocrine mechanism [15,41]. Our data evidenced lack of MSC engrafted in the myocardium and support a systemic effect of MSC in this rat OSA-model.

The development of atrial fibrosis in this OSA model might have important clinical implications if confirmed in humans. First, it demonstrates that pro-arrhythmogenic remodeling can be directly caused by OSA and not limited to remodeling induced by other comorbidities such as hypertension, obesity, coronary artery disease, or diabetes, all of which are very prevalent in OSA patients [21]. Accordingly, OSA screening might be important in AF-patients in order to establish a cause for the arrhythmia. Second, it may provide relevant prognostic information, as both OSA and atrial fibrosis predict poor outcomes of AF treatments. Finally, as OSA is a treatable disorder, early detection and treatment by means of CPAP or newer therapies may slow AF substrate progression and improve outcomes of antiarrhythmic drugs, electrical cardioversion, or AF ablation. Our results also suggest that MSC might have the potential to prevent the atrial profibrotic remodeling induced by OSA, but its clinical implication in humans needs to be demonstrated.

### Limitations

Some limitations should be acknowledged and taken into consideration when interpreting our results. First, we lack of electrophysiological and AF inducibility studies and thus, increased inducibility cannot be ensured from our results. However, we show that OSA induces atrial fibrosis, a hallmark of AF and a well-known AF promoter [8], at similar intensity to other reports showing increased inducibility [42]. Second, we assessed protein levels for some genes, including collagen deposit, inflammatory cytokines and MMP-2, but failed to obtain blots for ACE. Third, we only assessed atrial fibrosis as AF substrate; additional studies focusing on atrial dilation or electrical remodeling are warranted.

## Conclusions

In conclusion, our model demonstrates that OSA induces the development of atrial fibrosis, an arrhythmogenic substrate that might explain the association between OSA and atrial fibrillation. Fibrosis might be mediated by increased atrial expression of IL-6 and decreased atrial expression of MMP-2 with the subsequent decline in collagen-degradation. This atrial fibrosis is prevented by the intravenous administration of MSC, which normalizes IL-6 and MMP-2 expression.

## Competing interests

The authors declare that they have no competing interests.

## Authors’ contributions

LM, IA, JMM, JB, MB and PR: Conceptualized and designed the study. RF, JMM, MT, and IA: Developed and carried out OSA murine model. PR, CR and MB: Performed histological, plasma, mRNA and protein analysis and data interpretation. PR: Performed statistical analysis and wrote the first draft. All authors read and approved the final manuscript.
